# Arterial CO_2_ Fluctuations Modulate Neuronal Rhythmicity: Implications for MEG and fMRI Studies of Resting-State Networks

**DOI:** 10.1523/JNEUROSCI.4263-15.2016

**Published:** 2016-08-17

**Authors:** Ian D. Driver, Joseph R. Whittaker, Molly G. Bright, Suresh D. Muthukumaraswamy, Kevin Murphy

**Affiliations:** ^1^Cardiff University Brain Research Imaging Centre, School of Psychology, Cardiff University, Cardiff CF10 3AT, United Kingdom,; ^2^Sir Peter Mansfield Imaging Centre, Clinical Neurology, School of Medicine, University of Nottingham, Nottingham NG7 2RD, United Kingdom, and; ^3^Schools of Pharmacy and Psychology, Auckland University, Auckland 1142, New Zealand

**Keywords:** cortical oscillations, functional connectivity, hypercapnia, magnetoencephalography, physiological noise

## Abstract

A fast emerging technique for studying human resting state networks (RSNs) is based on spontaneous temporal fluctuations in neuronal oscillatory power, as measured by magnetoencephalography. However, it has been demonstrated recently that this power is sensitive to modulations in arterial CO_2_ concentration. Arterial CO_2_ can be modulated by natural fluctuations in breathing pattern, as might typically occur during the acquisition of an RSN experiment. Here, we demonstrate for the first time the fine-scale dependence of neuronal oscillatory power on arterial CO_2_ concentration, showing that reductions in alpha, beta, and gamma power are observed with even very mild levels of hypercapnia (increased arterial CO_2_). We use a graded hypercapnia paradigm and participant feedback to rule out a sensory cause, suggesting a predominantly physiological origin. Furthermore, we demonstrate that natural fluctuations in arterial CO_2_, without administration of inspired CO_2_, are of a sufficient level to influence neuronal oscillatory power significantly in the delta-, alpha-, beta-, and gamma-frequency bands. A more thorough understanding of the relationship between physiological factors and cortical rhythmicity is required. In light of these findings, existing results, paradigms, and analysis techniques for the study of resting-state brain data should be revisited.

**SIGNIFICANCE STATEMENT** In this study, we show for the first time that neuronal oscillatory power is intimately linked to arterial CO_2_ concentration down to the fine-scale modulations that occur during spontaneous breathing. We extend these results to demonstrate a correlation between neuronal oscillatory power and spontaneous arterial CO_2_ fluctuations in awake humans at rest. This work identifies a need for studies investigating resting-state networks in the human brain to measure and account for the impact of spontaneous changes in arterial CO_2_ on the neuronal signals of interest. Changes in breathing pattern that are time locked to task performance could also lead to confounding effects on neuronal oscillatory power when considering the electrophysiological response to functional stimulation.

## Introduction

The ability to detect and study human brain resting-state networks (RSNs) noninvasively *in vivo* is one of the great success stories of functional brain imaging techniques. The most widely adopted modality for studying RSNs is functional magnetic resonance imaging (fMRI). Although popular due to their finer spatial specificity, fMRI-based methods are not a direct measurement of local neuronal state, but rather a measurement of the local vascular response, sampling the blood vessels draining the neuronal populations of interest (Buxton, 2013). These fMRI-based methods are sensitive to contributions from physiological confounds such as the cardiac and respiratory cycles, blood pressure, and arterial CO_2_ concentration and, increasingly, these factors are monitored during data acquisition to apply correction techniques (Murphy et al., 2013). Electrophysiology-based methods for studying RSNs offer a potential advantage over fMRI in that they measure neuronal currents directly. Electroencephalography (EEG) (Laufs et al., 2003; Mantini et al., 2007) and magnetoencephalography (MEG)-based (Liu et al., 2010; de Pasquale et al., 2010; Brookes et al., 2011a, 2011b; Hipp et al., 2012) methods have been proposed recently, showing spontaneous oscillatory power modulations that are synchronized across functionally organized brain regions. These approaches have been applied to demonstrate dynamic cross-network integration (de Pasquale et al., 2012), to compare electrophysiological and hemodynamic measurements of RSNs (Hipp and Siegel, 2015), and to study the modulation of RSNs during natural vision stimulation (Betti et al., 2013), cognitive training (Astle et al., 2015) or pharmacological intervention (Muthukumaraswamy et al., 2013, 2015). Unlike fMRI-based RSN methods, electrophysiology-based RSN methods are generally assumed to be less sensitive to physiological modulations, so accompanying physiological measurements are not usually acquired or reported and it has been assumed that modulations in arterial CO_2_ do not affect MEG and EEG measurements. In this study, we challenge that assumption by showing that small natural fluctuations in arterial CO_2_, which occur during an RSN experiment, modulate neuronal oscillatory power significantly, thus potentially confounding MEG- and EEG-based RSN measurements.

Acute hypercapnia, an increase in arterial CO_2_ concentration, has been shown to reduce spontaneous neuronal oscillatory power in studies in anesthetized animals using intracortical electrodes (Jones et al., 2005; Zappe et al., 2008) and in awake humans using EEG (Bloch-Salisbury et al., 2000; Xu et al., 2011; Wang et al., 2015) and MEG (Hall et al., 2011). Reductions in spontaneous alpha power and increases in delta power have been observed during hypercapnia with EEG (Xu et al., 2011; Wang et al., 2015), whereas power reductions in alpha-, beta-, and gamma-frequency bands have been observed with MEG (Hall et al., 2011) and intracortical electrodes (Zappe et al., 2008). Because arterial blood gas measurements are invasive, end-tidal pCO_2_ (P_ET_CO_2_), the CO_2_ gas partial pressure at the end of exhalation, is typically used as a surrogate measurement of arterial CO_2_. Studies investigating the electrophysiological response to hypercapnia have used increases of 7–10 mmHg ΔP_ET_CO_2_ above the baseline normocapnic condition. These moderate levels of hypercapnia lie within the physiological range of P_ET_CO_2_ values observed during sleep, exercise, or at high altitude (Richerson, 2004; Ainslie and Duffin, 2009) and are of the range typically observed with 5% inspired CO_2_. However, P_ET_CO_2_ will also vary naturally over a range of 2–3 mmHg and a timescale in excess of 10 s due to spontaneous fluctuations in breathing pattern (Modarreszadeh and Bruce, 1994; Van den Aardweg and Karemaker, 2002; Wise et al., 2004). Here, we investigated whether these milder P_ET_CO_2_ modulations are sufficient to modulate oscillatory power significantly, consistent with recent findings that alpha power correlates with fluctuations in respiration (Yuan et al., 2013), and assess whether P_ET_CO_2_-induced modulations in neuronal oscillatory power could confound RSN measurements. We investigated the dependence of MEG oscillatory power on arterial CO_2_ both during a graded hypercapnia paradigm and in spontaneous P_ET_CO_2_ fluctuations to bridge the gap between previous studies using moderate hypercapnia and the smaller P_ET_CO_2_ fluctuations occurring during natural spontaneous changes in respiration.

## Materials and Methods

### 

#### 

##### Subjects.

Ten healthy subjects (age 30 ± 4 years, 3 female/7 male) participated in this study. The School of Psychology Cardiff University Ethics Committee approved this study and subjects gave informed consent before participating.

##### MEG acquisition.

Whole-head MEG recordings were made using a CTF 275-channel radial gradiometer system at a 600 Hz sampling rate. Four of the 275 channels were turned off due to excessive sensor noise. Participants were fitted with three electromagnetic head coils (nasion and preauriculars), which were localized relative to the MEG system throughout the data acquisition (10 Hz sampling frequency) to monitor head motion. For source localization, a T_1_-weighted anatomical MRI scan was acquired using a 3 T whole-body MRI system (GE Excite HDx) using an eight-channel receive coil. The anatomical scan was acquired using a 3D fast spoiled gradient recalled sequence with 1 mm isotropic voxel resolution covering the whole head.

##### Respiratory paradigm and physiological monitoring.

Gas mixtures were delivered to the subject through a tight-fitting face-mask (Quadralite; Intersurgical). Flow rates of two gas mixtures, medical air (21% O_2_, 79% N_2_) and a 5% CO_2_ mixture (5% CO_2_, 20% O_2_, 75% N_2_), were adjusted manually to provide an inspired gas mixture of 30 L/min. The respiratory circuit included a reservoir on the expired limb to permit rebreathing in the event that the instantaneous inspiratory rate exceeded 30 L/min. Expired gas concentrations were sampled from the face mask and P_ET_CO_2_ and end-tidal pO_2_ (P_ET_O_2_) were measured using rapidly responding gas analyzers (AEI Technologies).

**Figure 1.**
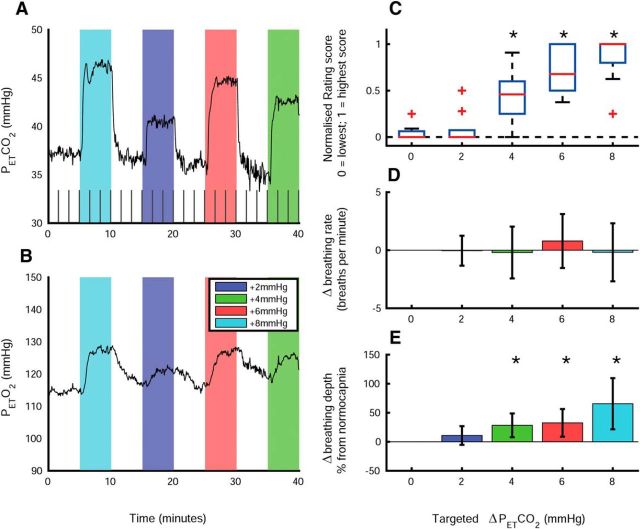
Physiological measures in response to graded hypercapnia. Shown are example P_ET_CO_2_ (***A***) and P_ET_O_2_ time courses (***B***), with color areas showing hypercapnia periods. N.B. the order of the four hypercapnia levels (+2, +4. +6 and +8 mmHg above baseline) were randomized across subjects. Each color represents a different hypercapnia level, so the color order (left–right) differs in this plot compared with subsequent plots in which hypercapnia level are displayed increasing sequentially. Gray boxes at the bottom of ***A*** indicate periods of the breathlessness rating feedback task. ***C***, Box plot of ratings feedback scores (red line, median across subjects; blue box, interquartile range; dashed whiskers, data range; red crosses, outliers). ***D***, Breathing rate change compared with the normocapnia (0 mmHg) level (mean ± SD across subjects). ***E***, Breathing depth percentage change compared with the normocapnia (0 mmHg) level (mean ± SD across subjects). Asterisks denote a significant change with respect to normocapnia (Bonferroni-corrected *p* < 0.05) based on a *post hoc* Wilcoxon signed-rank test for the discrete ratings feedback scores and a two-tailed paired *t* test for breathing rate and depth.

Each subject was presented with 4 levels of hypercapnia targeted at +2/+4/+6/+8 mmHg ΔP_ET_CO_2_ above their normal resting level. A manual feedback procedure was used to reach each hypercapnia level during which the respective flow rates of medical air and the 5% CO_2_ mixture were adjusted to reach the P_ET_CO_2_ target. Each level was maintained for 5 min and was preceded by a 5 min normocapnia period during which medical air was supplied at 30 L/min. The order of hypercapnia levels was randomized across subjects and each level was presented once per subject, giving a total protocol time of 40 min, as shown in Figure 1*A*.

In addition to P_ET_CO_2_, the following additional physiological measures were made to monitor the response to each hypercapnic level. Respiratory bellows were fitted to record chest motion for breathing rate and depth measurements. Subjective sensory feedback was acquired from the participants to test the hypothesis proposed by Hall et al. (2011) that there could be a significant sensory component to the changes in oscillatory power due to a conscious breathless sensation induced by the hypercapnia. Participants were asked to complete a breathlessness feedback rating task throughout the experiment, in which they were asked to rate the breathless sensation within the range of no breathlessness (“nothing”) to the breathlessness that they would expect to experience if they had been sprinting for a sustained period of time (“extreme”). This task was visually cued and participants responded by button press (right hand), moving a cursor along an incremental scale. The task was presented for 10 s in every 100 s (timings shown in Fig. 1*A*) while participants were asked to focus on a fixation point for the remaining 90 s. Due to the subjective nature of the task and the possibility of scaling bias across participants, the scores for each participant were scaled to range between 0 and 1, corresponding to the minimum and maximum score recorded by the participant, respectively. These normalized scores were used for statistics across the group, providing a subjective rating of the sensory response to hypercapnia.

##### Data analysis.

A 1–150 Hz band-pass filter was applied to the MEG dataset. The dataset was divided into 2 s trials that were visually inspected; those trials with gross artifacts (muscle artifacts, head movements, noise spikes) were discarded from subsequent analysis. Ten trials (20 s of data) were discarded for each feedback task to ensure that responses due to task performance did not affect the analysis of responses to changes in P_ET_CO_2_. This discarded period extended from 2 s before the onset of the feedback task to 8 s after the end of the task.

Source localization was performed on the remaining dataset using the beamformer algorithm synthetic aperture magnetometry (SAM) (Robinson and Vrba, 1998). The same source localization pipeline was performed as described in more detail previously (Muthukumaraswamy et al., 2013) and summarized as follows. A multiple local-spheres forward (Huang et al., 1999) volume conductor model was derived by fitting spheres to the brain surface extracted by the FSL Brain Extraction Tool (FSL, fMRIB, Oxford, UK; Smith, 2002) from the anatomical MRI scan. Global covariance matrices were calculated for the following 6 band-pass-filtered versions of the MEG dataset: delta (1–4 Hz), theta (4–8 Hz), alpha (8–13 Hz), beta (13–30 Hz), low gamma (30–50 Hz), and high gamma (50–90 Hz). Based on these covariance matrices, using the SAM algorithm (Robinson and Vrba, 1998), a set of beamformer weights was computed for all voxels in the brain at 8 mm isotropic voxel resolution. Virtual sensors were constructed at each voxel and the resultant time courses were normalized by an estimate of the projected noise amplitude at that voxel. The Hilbert transform was applied to each voxel time course and the absolute value was computed to generate an amplitude envelope of the oscillatory signals in each frequency band. The data at each voxel was down-sampled to 1 s epochs. This Hilbert amplitude dataset was realigned to standard (MNI152 atlas) space using FLIRT (Jenkinson et al., 2002).

Maps of the percentage change from normocapnia for each hypercapnia level were calculated as follows. The initial 100 s after a transition were discarded to avoid bias from transients (Hall et al., 2011). Mean values were calculated over each condition for each frequency band for each subject. The percentage change was calculated relative to the mean normocapnia Hilbert amplitude. To assess whether CO_2_ has a direct effect on Hilbert amplitude or if it is mediated by breathing depth and breathless sensation, mediation analysis was performed. A two-mediator model and bootstrapping (10000 bootstrap resamples), was implemented using the INDIRECT macro for SPSS (Preacher and Hayes, 2008). For each factor, mean values across each condition were inputted into the mediation model, with P_ET_CO_2_ as the independent variable, band-pass (alpha, beta, gamma) Hilbert amplitude as the dependent variable, and breathing depth and breathlessness score assessed as potential mediators.

Head motion can cause changes in SNR due to changing the distance between the source and sensors (Stolk et al., 2013). The possibility of the changes in Hilbert amplitude being due to motion correlated with changes in breathing pattern as opposed to the hypercapnia itself was assessed. Head localization measurements for the nasion and preauricular coils were each converted from a position vector to relative distance amplitude from the position at the start of the scan. This relative displacement amplitude was compared with P_ET_CO_2_ and Hilbert amplitude, as detailed in the Results section.

To further determine the behavior of neuronal oscillatory power under hypercapnia, the coordinates of peak alpha, beta, and low gamma amplitude decrease across the group for the +8 mmHg level (MNI coordinates: −14, −86, 32 mm alpha; 42, −14, 64 mm beta; 50, −6, 48 low gamma) were registered to each individual subject's native space and a virtual sensor constructed at each location (SAM, 1–100 Hz broadband beamformer weights). Frequency spectra were calculated for each 2 s trial using a multitaper frequency analysis method (FieldTrip, Donders Institute; Mitra and Pesaran, 1999; Oostenveld et al., 2011) with Slepian tapers and ± 2 Hz smoothing window.

## Results

Four well resolved levels of hypercapnia were achieved by increasing inspired CO_2_, targeted at +2/+4/+6/+8 mmHg ΔP_ET_CO_2_ above the normal resting level. Measured respiratory physiological responses to each level of hypercapnia are presented in Figure 1 and Table 1. Although the achieved ΔP_ET_CO_2_ slightly exceeded targeted values, each level was clearly resolved. Breathlessness feedback ratings scores, breathing rate, and depth for each level are plotted in Figure 1, *C–E*. Two statistical tests were performed: a repeated-measures ANOVA treating each ΔP_ET_CO_2_ level separately and a linear regression taking into account the measured variance in ΔP_ET_CO_2_ across subjects. The repeated-measures ANOVA tested each level (including the normocapnia level) as a discrete level, ignoring differences in ΔP_ET_CO_2_ across subjects. This allowed for *post hoc* tests to compare each level with the normocapnia level (Bonferroni corrected for multiple comparisons). The linear regression incorporated the actual ΔP_ET_CO_2_ measurements necessary for the mediation analysis described later. Repeated-measures ANOVA and linear regression results are presented in Table 2. Breathing depth and normalized feedback rating scores showed a significant dependence on ΔP_ET_CO_2_, whereas breathing rate did not.

**Table 1.** Measured respiratory physiological response to the graded hypercapnia levels

Targeted ΔP_ET_CO_2_ (mmHg)	+2 mmHg	+4 mmHg	+6 mmHg	+8 mmHg	Normocapnia absolute value
Achieved ΔP_ET_CO_2_ (mmHg)	3.4 ± 1.1*	5.9 ± 1.0*	7.2 ± 1.3*	9.3 ± 0.9*	37 ± 2
ΔP_ET_O_2_ (mmHg)	3.3 ± 2.3*	5.5 ± 3.0*	8.1 ± 1.8*	8.7 ± 3.4*	118 ± 2
Δ breathing rate (breaths/min)	−0.0 ± 1.3	−0.2 ± 2.2	0.8 ± 2.3	−0.2 ± 2.5	13 ± 3
% change in breathing depth	10.8 ± 16.1	28.3 ± 20.5*	32.5 ± 23.9*	65.5 ± 44.1*	N/A

Values presented are mean ± SD across subjects of the change relative to normocapnia. The right column shows absolute values at normocapnia. Note: The breathing depth measurement is based on chest motion and is not calibrated, so only provides relative measurements.

*Significant change with respect to normocapnia (Bonferroni-corrected *p* < 0.05). N/A, Not applicable.

**Table 2.** Repeated-measures ANOVA and linear regression statistics

	Repeated measures ANOVA*^a^*			Linear regression with ΔP_ET_CO_2_*^b^*		
	df_1_/df_2_	*F*	*p*	*r*	*F*_(1,38)_	*p*
Breathing rate	1.5/13.7	0.5	0.6	0.16	1.0	0.3
Breathing depth*	1.5/13.1	9.8	0.004	0.57	18.1	<0.001
Feedback rating*	4/36	25.5	<0.001	0.64	26.2	<0.001
Head motion*^c^*						
Mean across block	2.2/19.6	1.98	0.2	0.11	0.5	0.5
SD across block	1.9/17.2	25.7	<0.001	0.08	0.3	0.6
Hilbert amplitude						
Delta (1–4 Hz)	2.1/18.9	0.7	0.5	0.03	0.0	0.8
Theta (4–8 Hz)	4/36	1.3	0.3	0.13	0.7	0.4
Alpha (8–13 Hz)*	4/36	6.2	0.001	0.33	4.7	0.04
Beta (13–30 Hz)*	4/36	17.4	<0.001	0.61	22.3	<0.001
Low gamma (30–50 Hz)*	4/36	10.1	<0.001	0.53	15.1	<0.001
High gamma (50–90 Hz)	4/36	0.9	0.5	0.21	1.7	0.2

Hilbert amplitudes were averaged across the whole brain and then across each condition.

*^a^*The ANOVA tested for significant differences across the five conditions (normocapnia and four hypercapnia levels).

*^b^*The linear regression tested for a significant dependence on ΔP_ET_CO_2_, rather than treating each hypercapnia level as a discrete condition.

*^c^*Head motion parameters presented here were formed by taking the median value from nasion and preauricular coils (after calculating either mean or SD across each condition.

*p < 0.05 for both tests.

### Oscillatory amplitude decreases with increasing inspired CO_2_

**Figure 2.**
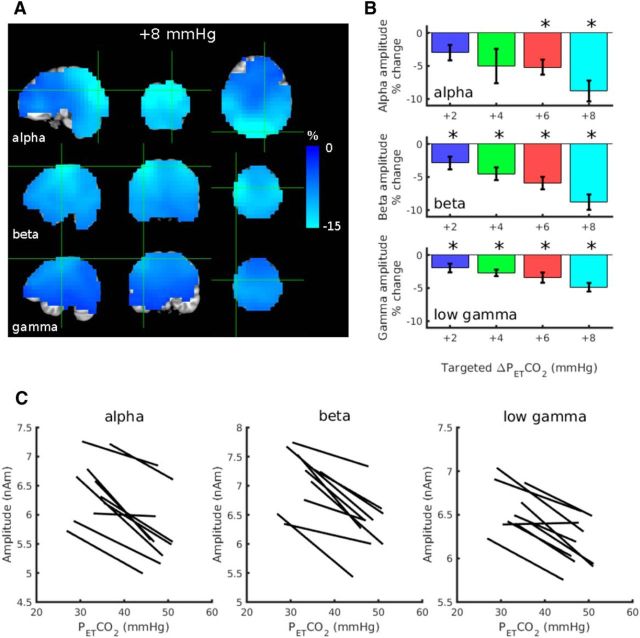
The MEG oscillatory amplitude response to hypercapnia. ***A***, Group average Hilbert amplitude response percentage change maps for alpha, beta, and low-gamma bands for the highest (+8 mmHg target) level of hypercapnia relative to normocapnia (*p*_corr_ < 0.01, 2-tailed *t* test across subjects). ***B***, Whole-brain average percentage change in alpha, beta, and gamma amplitude (mean ± SEM across subjects) for each hypercapnia level (relative to the medical air breathing normocapnia periods). Asterisks denote a significant change from normocapnia (Bonferroni-corrected *p* < 0.05, *post hoc* 2-tailed *t* test). ***C***, Line of best fit for each subject (1 line per subject) between whole-brain average Hilbert amplitude and P_ET_CO_2_ on an epoch-wise basis. Example scatter plots that these lines of best fit are based on are presented later in Figures 4 and 5.

Neuronal oscillatory amplitudes in the delta (1–4 Hz), theta (4–8 Hz), alpha (8–13 Hz), beta (13–30 Hz), low gamma (30–50 Hz), and high gamma (50–90 Hz) frequency bands were assessed for potential changes with hypercapnia. Before assessing the responses across graded levels, consistency with the results reported previously by Hall et al. (2011) was assessed in Figure 2*A*, showing group average percentage change Hilbert amplitude maps for the highest (+8 mmHg target) level of hypercapnia, for alpha, beta, and low-gamma bands. The spatial characteristics and amplitudes observed here are similar to those reported in that study (Hall et al., 2011).

Considering the responses across hypercapnia levels, the percentage change in Hilbert amplitude averaged across the whole brain is presented in Figure 2*B* for each hypercapnia level (relative to normocapnia), for alpha, beta, and low-gamma bands. No significant changes in Hilbert amplitude were observed in the delta, theta, and high-gamma bands. Repeated-measures ANOVA and linear regression results are presented for each frequency band in Table 2. Further, making an assumption of a linear relationship between whole-brain average Hilbert amplitude and P_ET_CO_2_, lines of best fit are plotted for each subject in Figure 2*C*, showing negative relationships in all subjects for alpha and beta band and for all but one subject in the low-gamma band.

To evaluate how head motion might affect the block-averaged Hilbert amplitudes calculated for each hypercapnia level, both mean and SD of head motion relative distance over each hypercapnia level was compared with ΔP_ET_CO_2_ and each of the MEG frequency band amplitudes. Neither mean nor SD of head motion estimates correlated with ΔP_ET_CO_2_ or delta, theta, alpha, beta, or low-gamma frequency band amplitudes when averaged across each hypercapnia level. High-gamma band amplitude showed a positive correlation with head motion (*r* = 0.32; *F*_(1,38)_ = 4.2; p = 0.05). There was a significant (*p* < 0.001) effect of hypercapnia level on SD of head motion across each level (repeated-measures ANOVA). Repeated-measures ANOVA and linear regression results for head motion are shown in Table 2 for the median values across the nasion and preauricular coils.

With significant relationships between breathing depth and feedback scores (subjective breathlessness) with ΔP_ET_CO_2_ level, the question arose of whether the observed changes in alpha, beta, and low-gamma band amplitude are due directly to the hypercapnic stimulus or if they are mediated by the breathing depth or breathlessness. Results for mediation analysis, testing breathlessness and breathing depth as mediators of oscillatory amplitude, are presented in Table 3. This analysis suggests that breathing depth is a mediator of the changes in alpha-band amplitude, but otherwise breathlessness and breathing depth do not mediate the Hilbert amplitude decreases observed significantly. Note, however, that interpretation of the mediation analysis is limited by the relatively small sample size of this study compared with the social psychology datasets for which it was originally designed. Despite this, our results suggest that sensory perception of the inspired CO_2_ challenge does not contribute significantly to the observed reductions in alpha, beta, and gamma amplitudes. This is further supported by only considering the mildest hypercapnia level (+2 mmHg target) at which significant reductions in beta and low-gamma amplitude and a trend in alpha amplitude were observed despite no significant increase in the breathlessness rating compared with normocapnia.

**Table 3.** Mediation analysis results

	Alpha band	Beta band	Low gamma
Simple model			
Direct effect	−0.747	−0.925	−0.481
Mediator model			
Direct effect	−0.329	−0.696	−0.347
Indirect effects			
Total	−0.458 [−1.169, 0.331]	−0.260 [−0.680, 0.216]	−0.144 [−0.424, 0.131]
Feedback rating	−0.072 [−0.458, 0.235]	−0.054 [−0.458, 0.235]	−0.096 [−0.347, 0.095]
Breathing depth	−0.387* [−0.814, −0.001]	−0.206 [−0.482, 0.034]	−0.048 [−0.249, 0.106]

Regression coefficients (units of %/mmHg) [bootstrap 95% confidence intervals]. The simple model is a linear regression between ΔP_ET_CO_2_ and Hilbert amplitude at each frequency band. The mediator model also includes breathlessness (normalized feedback rating score) and breathing depth as potential mediators. Indirect effects were tested using a bootstrapped sampling distribution, with significance determined by zero not being included within the 95% confidence intervals. Cases where this condition is met are marked with an asterisk.

**Figure 3.**
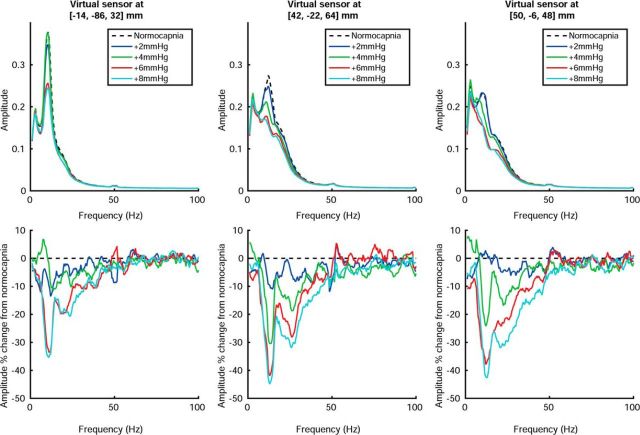
Top, Frequency spectra for each condition for virtual sensors positioned at the peak response coordinate for alpha (left), beta (middle), and low-gamma (right) bands. MNI coordinates for these virtual sensors are presented in the title of each plot. Bottom, The same spectra plotted as an amplitude percentage change from medical air (normocapnia) periods for each hypercapnia level.

Figure 3 demonstrates frequency spectra (see last paragraph of Materials and Methods) averaged across trials within a condition and then across subjects. There is a graded broadband reduction in oscillatory amplitude across alpha, beta, and gamma bands with hypercapnia level, but also a pronounced suppression of alpha and beta peaks. No shift in oscillatory power between frequency bands was observed, unlike for task-evoked responses, for which reduced power in one frequency band is typically accompanied by an increased power in another frequency band (Mukamel et al., 2005; Zumer et al., 2010).

### Spontaneous fluctuations in P_ET_CO_2_ correlate with oscillatory amplitude

**Figure 4.**
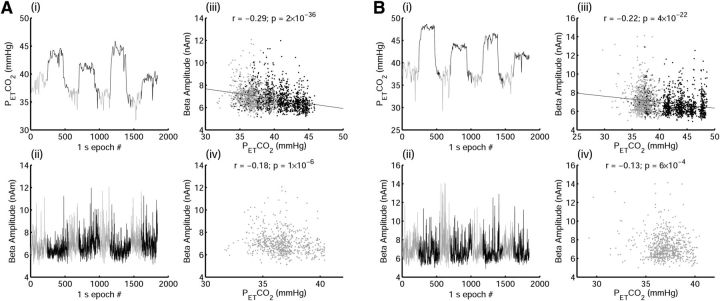
Spontaneous fluctuations in P_ET_CO_2_ during periods of medical air and corresponding beta-band amplitude demonstrated in a subject with a high correlation coefficient (***A***) and a subject with a median correlation coefficient (***B***). ***i***, P_ET_CO_2_ time series with normocapnia periods shown in gray. ***ii***, Whole-brain average beta amplitude time series. ***iii***, Plot of beta amplitude against P_ET_CO_2_ for each time point across the whole time series with corresponding Pearson correlation results displayed above the plot and the line of best fit across the whole time series plotted in black. ***iv***, Plot of beta amplitude against P_ET_CO_2_ for normocapnia time points only with corresponding Pearson correlation results.

**Figure 5.**
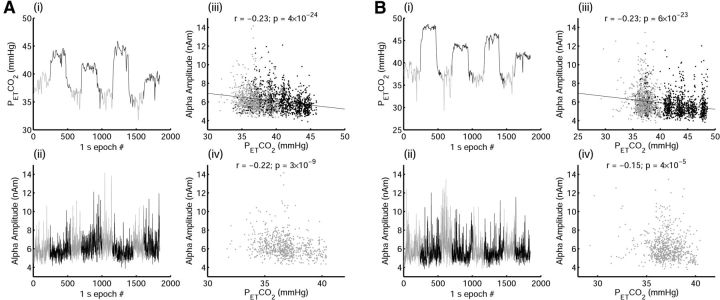
Spontaneous fluctuations in P_ET_CO_2_ during periods of medical air and corresponding alpha-band amplitude demonstrated in a subject with a high correlation coefficient (***A***) and the subject with a median correlation coefficient (***B***). ***i***, P_ET_CO_2_ time series with normocapnia periods shown in gray. ***ii***, Whole-brain average alpha amplitude time series. ***iii***, Plot of alpha amplitude against P_ET_CO_2_ for each time point across the whole time series with corresponding Pearson correlation results displayed above the plot and the line of best fit across the whole time series plotted in black. ***iv***, Plot of alpha amplitude against P_ET_CO_2_ for normocapnia time points only, with corresponding Pearson correlation results.

With significant reductions in oscillatory amplitude observed even at the mildest level of hypercapnia, the question arises as to whether changes in oscillatory amplitude can be observed during spontaneous fluctuations in PaCO_2_ or if these changes are restricted to the controlled CO_2_ inhalation stimulus. To investigate this, we selected the normocapnia periods during which medical air was supplied and compared the P_ET_CO_2_ trace for these periods with the Hilbert amplitude at each frequency band. The initial 100 s of normocapnia after a hypercapnia challenge was discarded to ensure that transients from preceding gas periods did not affect this analysis (Hall et al., 2011). The intrasubject SD of P_ET_CO_2_ across the remaining normocapnia periods was 1.5 mmHg (median across subjects, range 0.9–3.8 mmHg). Epoch-wise comparison was made between whole-brain Hilbert amplitude and P_ET_CO_2_, assuming independence between each 1 s epoch of MEG data. Demeaned whole-brain Hilbert amplitude showed significant epoch-wise dependence on these spontaneous P_ET_CO_2_ fluctuations for delta, alpha, beta, and low-gamma bands (fixed-effects general linear model), as reported in Table 4. The fixed-effects general linear model is a conservative approach suitable for assessing significance, but it can underestimate *R* values due to intersubject variability. Therefore, results for a random-effects general linear model are also presented in Table 4. These show *R* values of ∼0.13–0.15 (i.e., P_ET_CO_2_ fluctuations accounting for 1.7–2.2% of the signal variance). This relationship was further interrogated at an individual subject level (Table 5) by calculating the Pearson correlation coefficient between P_ET_CO_2_ and Hilbert amplitude across normocapnia epochs. To evaluate any potential effect of head motion in these epoch-wise analyses, a partial correlation was also performed, providing a partial *r* for the effect of P_ET_CO_2_ on Hilbert amplitude after removing the effect of head motion. In this partial regression, head motion relative distance was averaged within each 1 s epoch and nasion and preauricular coils were treated as separate columns in the correlation. Mean correlation coefficients and summary statistics across subjects (after Fisher *z*-transformation) are presented in Table 5. The statistic presented is a 2-tailed *t* test across subjects, testing Fisher *z* ≠ 0 at a *p* < 0.05 significance level. Negative correlations between Hilbert amplitude and P_ET_CO_2_ were observed in alpha and beta bands both with and without accounting for head motion, whereas significant negative correlations were only observed in delta, theta, and low-gamma bands in the partial correlation after accounting for head motion. This negative correlation is demonstrated in the beta band for both the subject with a high correlation coefficient and the subject with the median correlation coefficient in Figure 4 and for the alpha band in Figure 5.

**Table 4.** Group-level fixed-effects and random-effects general linear model results

Frequency band	*R*	*F*	*p*	Amplitude (%/mmHg)
Fixed effects				
*Delta (1–4 Hz)	0.07	38.5	6 × 10^−10^	−0.59*
Theta (4–8 Hz)	0.02	1.7	0.19	−0.12
Alpha (8–13 Hz)*	0.09	57.8	3 × 10^−14^	−0.86*
Beta (13–30 Hz)*	0.06	26.8	2 × 10^−7^	−0.42*
Low gamma (30–50 Hz)*	0.06	26.5	3 × 10^−7^	−0.23*
High gamma (50–90 Hz)	0.02	2.0	0.16	−0.06
Random effects				
Delta (1–4 Hz)	0.15	15.2	3 × 10^−27^	−0.59
Theta (4–8 Hz)	0.13	12.0	6 × 10^−21^	−0.65
Alpha (8–13 Hz)	0.15	15.4	1 × 10^−27^	−1.23
Beta (13–30 Hz)	0.15	15.8	2 × 10^−28^	−0.84
Low gamma (30–50 Hz)	0.15	16.9	1 × 10^−30^	−0.41
High gamma (50–90 Hz)	0.14	14.5	8 × 10^−26^	0.21

Demeaned Hilbert amplitude tested against a model formed of spontaneous fluctuations in P_ET_CO_2_.

*p < 0.05

**Table 5.** Subject-level linear correlation results comparing the effect of spontaneous fluctuations in P_ET_CO_2_ on Hilbert amplitude for each frequency band

Frequency band	Correlation with P_ET_CO_2_ only, ignoring head motion parameters*^a^*		Partial correlation with P_ET_CO_2_, accounting for head motion parameters*^b^*	
	*r*	*p*	*r*	*p*
Delta (1–4 Hz)	−0.07	0.16	−0.09	0.04*
Theta (4–8 Hz)	−0.06	0.15	−0.07	0.05*
Alpha (8–13 Hz)	−0.11	0.01*	−0.07	0.02*
Beta (13–30 Hz)	−0.10	0.04*	−0.07	0.04*
Low gamma (30–50 Hz)	−0.08	0.09	−0.08	0.01*
High gamma (50–90 Hz)	0.04	0.26	−0.006	0.89

Correlation values are averaged across subjects after Fisher *z*-transformation.

*^a^*Pearson *r* values are presented when P_ET_CO_2_ considered in isolation of head motion.

*^b^*Partial *r* values are presented for P_ET_CO_2_, controlling for head motion.

*p < 0.05 based on Fisher *z* ÷ 0, two-tailed *t* test across subjects.

**Figure 6.**
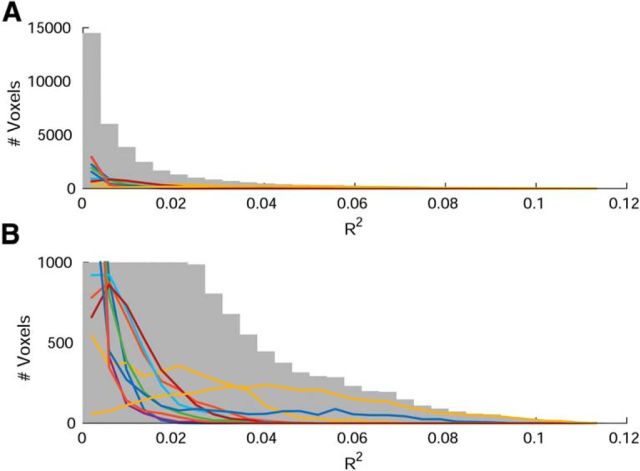
***A***, Histogram showing voxelwise *R*^2^ indicating the contribution of P_ET_CO_2_ fluctuations to the beta-amplitude signal. The gray histogram indicates all voxels across all subjects, whereas the lines indicate the histograms of voxels for each individual subject. ***B***, Windowed version of ***A*** to highlight the length of the tail and the shape of each individual subject histogram.

To estimate the voxelwise relative contribution of P_ET_CO_2_ fluctuations to the beta-band Hilbert amplitude signal, a voxelwise linear regression was made between P_ET_CO_2_ and beta amplitude. Histograms of *R*^2^ for this regression are shown in Figure 6 both for the summation of all voxels across all subjects (gray histogram) and for each subject individually (lines). These histograms demonstrate both intrasubject and intersubject variability in the distribution of variance explained by P_ET_CO_2_ fluctuations, ranging up to 10% (*R*^2^ = 0.1).

## Discussion

This study investigated the effect of arterial CO_2_ concentrations on MEG-based measurements of neuronal oscillatory power and the implications for electrophysiological measurements of RSNs. We showed that neuronal oscillatory power has a graded dependence on arterial CO_2_ concentration, with significant decreases in beta and gamma power extending to a very mild range of increases in arterial CO_2_, such as may be caused by spontaneous natural variations in breathing pattern. The graded hypercapnia design included finer steps in P_ET_CO_2_ (and arterial CO_2_ concentration) than have been used previously in electrophysiology studies. Further, we investigated neuronal oscillatory power in periods with no supplementary inspired CO_2_ and we provide the first data showing a direct correlation between spontaneous changes in P_ET_CO_2_ and neuronal oscillatory power. Our results suggest that spontaneous changes in P_ET_CO_2_ account for ∼2% (*R* ∼0.15; Table 4) of the resting-state MEG signal variance. To put this effect into perspective, seed-based resting-state MEG studies typically find maximal correlation coefficients of approximately *R* ∼0.3 between nodes of the same network (e.g., left and right motor cortices) and ∼10% or less shared signal variance between nodes (Brookes et al., 2011a; Brookes et al., 2011b; Hipp et al., 2012; Tewarie et al., 2014).

In the following paragraph, we consider how the data that we present here contribute to understanding of the mechanisms underlying the neuronal response to hypercapnia. A possible sensory cause has been proposed previously (Hall et al., 2011), suggesting that a conscious awareness of the altered inspired gas concentrations could be responsible. We were able to test this hypothesis by acquiring MEG data and a subjective feedback rating of breathlessness over a range of inspired CO_2_ concentrations. By investigating this relationship across the graded levels, our results demonstrate that this conscious awareness does not contribute significantly to the CO_2_-induced reduction in MEG oscillatory power. The most likely mechanism for the dependence of neuronal oscillatory power on arterial CO_2_ is neurochemical in origin through an inverse relationship between arterial CO_2_ concentration and pH (Kety and Schmidt, 1948; Ito et al., 2003). This is thought to be due to an inverse relationship between extracellular adenosine concentration and pH. Adenosine release acts to reduce cortical excitability during hypercapnia (Dulla et al., 2005). Our results are consistent with this extracellular pH-based mechanism, as the small arterial CO_2_ levels are unlikely to overcome pH buffering to modify intracellular pH. Another possible mechanism that deserves consideration is through arterial CO_2_ modulating brain temperature (Kauppinen et al., 2008). Heat generated through cerebral energy metabolism is dissipated by cerebral blood vessels such that arterial vasodilation and increased cerebral perfusion in the absence of increased metabolism (such as occurs during hypercapnia) will have a cooling effect on the brain (Sukstanskii and Yablonskiy, 2006). For a similar level of hypercapnia (ΔP_ET_CO_2_ = +8 mmHg) as we use for our highest level, brain temperature decreased by 0.3°C in the human occipital cortex (Kauppinen et al., 2008). The effect of such a mild temperature change on cerebral physiology is unclear (Kiyatkin, 2007), whereas the temperature changes due to spontaneous fluctuations in arterial CO_2_ will remain small and thus are unlikely to cause the neuronal oscillatory changes that we observed.

Our findings have implications for studies using spontaneous oscillatory power modulations to investigate RSNs (de Pasquale et al., 2010; Brookes et al., 2011b; Hipp et al., 2012). We suggest that future studies record P_ET_CO_2_ throughout data acquisition, as can be done without discomfort for the participant by use of a nasal cannula. We foresee that correction techniques already implemented for similar fMRI studies (Murphy et al., 2013) can be easily adapted to remove unwanted signal contributions due to P_ET_CO_2_ fluctuations. However, our findings do not discriminate whether these CO_2_-dependent oscillatory power modulations are incidental noise confounding measurements or an interesting interaction with the neuronal oscillations under investigation. Either way, these results also have implications for fMRI RSN studies, in which it is commonly assumed that signal fluctuations due to P_ET_CO_2_ are purely vascular in nature (Murphy et al., 2013), whereas our findings show that there is also a neuronal component that should be considered.

These results not only have implications for RSN studies, but also for task-evoked MEG studies. Complex task performance can lead to task-locked ventilation changes (Birn et al., 2009). This will lead to a perturbation in arterial CO_2_, which will modulate neuronal oscillatory power in a manner that is time locked to the task. Subsequent time–frequency analysis will therefore be confounded because this CO_2_-related modulation will not cancel out when averaging across trials. This noise contribution will have a large intersubject variability because the task-locked breathing pattern will differ across subjects and could be further emphasized when comparing across clinical populations or during pharmacological interventions, when respiration may be altered. In addition, task-evoked event-related potentials are attenuated during moderate hypercapnia (Bloch-Salisbury et al., 2000; Jones et al., 2005; Thesen et al., 2012), potentially adding to the confound. A recording of P_ET_CO_2_ during data acquisition could be used to characterize this noise contribution retrospectively.

We report a graded decrease in alpha, beta- and low-gamma band amplitude with increasing P_ET_CO_2_. The graded hypercapnia results show that the robust results seen previously (Hall et al., 2011) extrapolate to smaller ΔP_ET_CO_2_ increments (2–3 mmHg above baseline) of the order typical during spontaneous variations in respiration. Participant feedback of subjective breathlessness was combined with the graded design to consider the effect of the conscious sensory response, showing that it is not a major cause of the amplitude decreases observed with hypercapnia. A subconscious response to hypercapnia, namely breathing depth, appears to be a factor in the reduction of alpha-band amplitude. This is consistent with EEG findings showing correlations between alpha power and fluctuations in respiration (Yuan et al., 2013).

By only considering normocapnia periods, during which no CO_2_ was supplied in the inspired gas mixture, spontaneous fluctuations in P_ET_CO_2_ were shown to correlate with spontaneous fluctuations in delta-, alpha-, beta-, and gamma-band amplitude. This fine-scale effect has implications for both MEG and fMRI studies using resting-state signal fluctuations to identify neural networks. An interesting finding is that there is a significant relationship between delta power and spontaneous fluctuations in P_ET_CO_2_, but not with the graded hypercapnia P_ET_CO_2_. This could be consistent with modulation of delta power across the breathing cycle (Busek and Kemlink, 2005). With our data, we were unable to assess temporal lags between spontaneous arterial CO_2_ fluctuations and spontaneous neuronal fluctuations. Although the MEG data are well suited to considering finer timescales, the P_ET_CO_2_ data are limited to the period of the breathing cycle, so this experiment was unsuitable for resolving a subsecond timescale.

One aspect that we have not controlled for in this experiment is changes in the participant's alertness throughout the experiment. There is likely to be a low-order change in alertness across the 40 min experiment and alertness is likely to change breathing patterns and P_ET_CO_2_. Randomizing the order of presentation of each hypercapnia level across subjects controlled for the low-order change in alertness; however, alertness may be modulated by the hypercapnia challenge itself due to the mild discomfort caused by the breathless sensation at the higher levels of hypercapnia. We assessed this by recording a subjective measure of breathlessness and using mediation analysis, which suggested that the breathless sensation was not a significant mediator of the oscillatory amplitude decreases that we observed. Further, the same inverse relationship was shown between P_ET_CO_2_ and alpha, beta, and gamma amplitude when assessed both across graded levels of hypercapnia and during spontaneous fluctuations in the normocapnia periods. Alertness and P_ET_CO_2_ vary inversely during spontaneous variations in ventilation (Oken et al., 2006) because ventilation reduces and P_ET_CO_2_ increases as attention drifts. During hypercapnia, however, alertness is expected to increase at high levels of P_ET_CO_2_ in response to the breathless sensation. Therefore, the similar results in graded hypercapnia and spontaneous ventilation exclude alertness as a dominant contributor to the measured relationship between P_ET_CO_2_ and oscillatory amplitude.

The natural breathing cycle causes a small periodic head motion, which in this supine study, was ∼1 mm; however, our analyses suggest this did not drive the effects that we observed here. MEG signal SNR and source localization will be affected by changes in head position (relative to the sensors) across the breathing cycle, whereas arterial CO_2_ (and P_ET_CO_2_) is related to ventilation, which is integrated across the breathing cycle. Head motion was compared across hypercapnia levels by both absolute distance and variance in head motion across the block. There was no significant change in absolute distance (mean across block) between hypercapnia levels, showing no systematic shift in head position between hypercapnia levels. Head motion variance (SD across block) did increase across hypercapnia levels, consistent with increased breathing depth with increasing levels of hypercapnia. Interestingly, this effect disappeared when taking account of measured individual changes in P_ET_CO_2_ across each level. At the block level, this did not translate to a significant effect on Hilbert amplitude apart from in the high-gamma band. Spontaneous variations in breathing pattern are associated with a negative relationship between P_ET_CO_2_ and breathing depth, with increased ventilation (and, thus, motion) leading to reduction in arterial CO_2_ (Banzett et al., 2000). When correlating spontaneous fluctuations in P_ET_CO_2_ and Hilbert amplitude, the potential effects of head motion were removed through use of a partial correlation approach, observing significant negative correlations between P_ET_CO_2_ and Hilbert amplitude when only considering the components of each trace that are independent from head motion. Breathing depth (and the associated change in the amplitude of head motion oscillations) changes in opposite directions with P_ET_CO_2_ for the case in which supplemental CO_2_ is inspired (hypercapnia) and for the case of spontaneous variations in breathing pattern. Therefore, the observed negative dependence of Hilbert amplitude on P_ET_CO_2_ both with additional inspired CO_2_ and during spontaneous variations in breathing pattern rules out head motion being the major cause of the observed effect.

### Conclusions

In conclusion, we have considered the possible impact of respiration on electrophysiological measurements of human brain RSNs, demonstrating that small fluctuations in arterial CO_2_ caused by spontaneous fluctuations in breathing can lead to significant fluctuations in resting-state MEG power, causing a physiological noise source for MEG RSN experiments. The fine-scale relationship between arterial CO_2_ and neuronal oscillations is of significance for MEG, EEG, and fMRI studies, in which conclusions may be confounded by accompanying changes in arterial CO_2_-induced neuronal oscillatory power.
